# Chemical Analysis through CL-Detection Assisted by Periodate Oxidation

**DOI:** 10.1155/2007/92595

**Published:** 2007-03-25

**Authors:** Nicholaos P. Evmiridis, Athanasios G. Vlessidis, Nicholas C. Thanasoulias

**Affiliations:** Laboratory of Analytical Chemistry, Department of Chemistry, University of Ioannina, 451 10 Ioannina, Greece

## Abstract

The progress of the research work of the author and his colleagues on the field of CL-emission generated by pyrogallol oxidation and further application for the direct determination of periodate and indirect or direct determination of other compounds through flow-injection manifold/CL-detection set up is described. The instrumentation used for these studies was a simple flow-injection manifold that provides good reproducibility, coupled to a red sensitive photomultiplier that gives sensitive CL-detection. In addition, recent reports on studies and analytical methods based on CL-emission generated by periodate oxidation by other authors are included.

## 1. INTRODUCTION

During a chemical reaction process, the reactant atoms are combined in different ways to form new compounds called products. Apart from the rearrangement of the atoms involved in the reaction process, a change in energy occurs which either is released to the environment or taken from the environment. The energy released by a spontaneous reaction to the environment is in most cases heat energy measured in calories per mole and such reactions are called exothermic. An exothermic reaction may also release energy in other forms of energy apart from heat. Reactions that release energy greater than 41 kcal per mole can give CL-emission. Oxidation reactions are usually accompanied by large energy release. The wavelength of CL-emission is calculated from the equation

(1) $$\text{E}=\text{h}\frac{c}{\lambda }$$,

where *h* = Planck constant, *c* = the velocity of light, and *λ* = the wavelength of the emitted light.

The CL-intensity, on the other hand, is limited by the rules of the quantum yield, and the thermodynamic and kinetic constants of the reaction process as well as by the presence of quenching effects.

**Figure 1:**
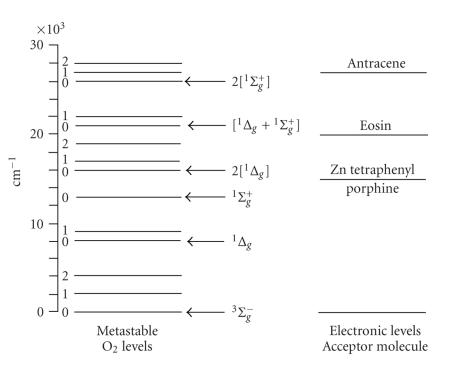
Electronic energy levels of molecular oxygen and excited singlet molecular oxygen dimmers available for energy transfer in chemiluminescence (horizontal arrows label the pure electronic states).

CL-reagents are in most cases light absorbing compounds of rigid structure in order to be deactivated by CL-emission when are activated from the oxidation reaction. If the activated species have very low quantum yield either because they relax with heat release or the environment favors quenching, a good advice is to add a fluorescent compound of relatively high yield in the reaction mixture to enable energy-transfer from the activated species to the fluorescent molecule. Such an example is the energy transfer of (O_2_)_2_^*^ excimer to fluorescent compound that has energy states close to that of the excimer [1, 2] as shown in Figure 1.

### 1.1. CL-reagents

**Figure 2:**
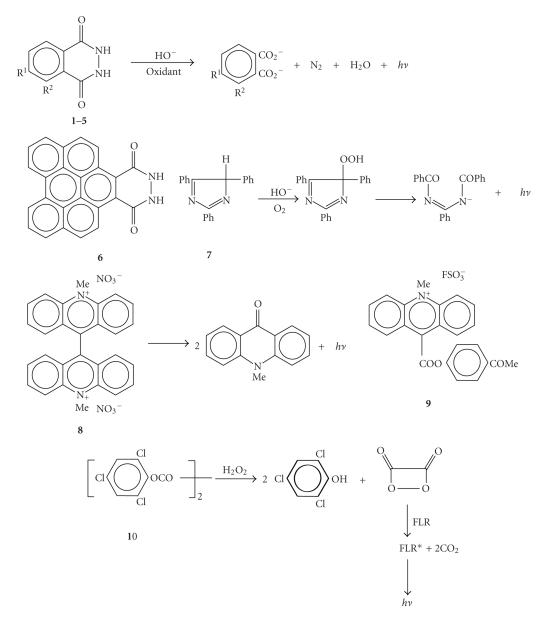
Chemiluminescent compounds and reactions. Formulae **1**–**5** are as follows: **1**, R^1^ = H, R^2^ = NH_2_; **2**, R^1^ = NH_2_, R^2^ = H; **3**, R^1^ = HO_2_CCH_2_CONH(CH_2_)_4_NC_2_H_5_, R^2^ = H; **4**, R^1^ = H_2_N(CH_2_)_6_NC_2_H_5_, R^2^ = H; **5**, R^1^ = H_2_N(CH_2_)_4_NC_2_H_5_, R^2^ = H.

The CL-reagents used in chemical analysis are mostly Luminol, Lucigenin, TCPO, and acridine derivatives. TCPO has the largest quantum yield (21%) from all while the other ones around 1–5%. All of them show rapid rate of reaction with oxidants [3]. The oxidation reactions that take place with the above CL-reagents are shown in Figure 2. Pyrogallol is a molecule that can be oxidized fairly easily by most of the oxidants, but with a faint only CL-emission [3].

### 1.2. Pyrogallol in human activities and its CL-detection in analytical chemistry

Pyrogallol or benzene-1,2,3-triol (C_6_H_6_O_3_) is a white crystalline powder and a powerful reducing agent. It is used in photography as developing agent, hair dying, dying of suturing materials, and for oxygen adsorption in gas analysis. It is also used in surgery for his antiseptic properties. The material is available as an analytical chemical reagent with a purity > 99%.

Pyrogallol structural group is found in polyphenols, tannins, as constituents of a great variety of various plant products, fruits, and nutrition products, for example, red wine, grapes, tea, and so forth. Humic acids include compounds that bear the pyrogallol or gallic acid functional group. Tannins are also used in various industrial processes for the treatment of various raw materials like leather.

### 1.3. Periodate oxidation of pyrogallol mechanism

Periodate oxidation of different methoxy derivatives of pyrogallol proceeds rapidly with the production of *o*- and *p*-quinones, and slowly with products of quinone condensation and the formation of Diels-Alder dimmers.

The complexity of periodate oxidation chemistry includes a wealth of products that are formed through different reaction pathways generated by the reactive quinone intermediates and are kinetically controlled. The condensed quinone products bear the necessary requirements for an energy transfer process from excited species since these molecules include double bonds and their structure is rigid; and they present a tempting case of investigation possibilities of enhancing CL-emission.

**Figure 3:**
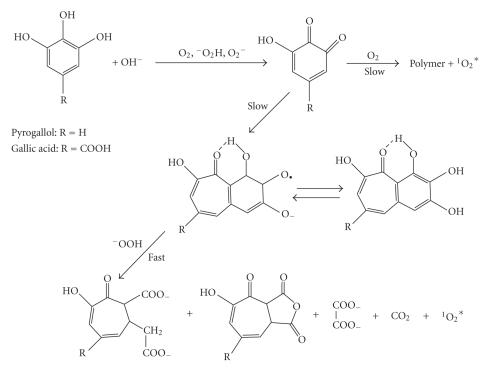
Trazt-Schorigin reaction for the oxidation of pyrogallol by hydrogen peroxide.

An example of the mechanism of pyrogallol oxidation by hydrogen peroxide through the so-called Trazt-Schorigin reaction [4–6] is given in Figure 3.

During this oxidation process, an excited singlet oxygen is formed and the light emission occurs via intermolecular energy transfer as well as simple excimer emission according to reaction,

(2) (^1^O^*^_2_)_2_ + P → 2^3^O_2_ + P^*^ → P + *hv*
(475–505 nm).

### 1.4. Oxygen equilibria

**Figure 4:**
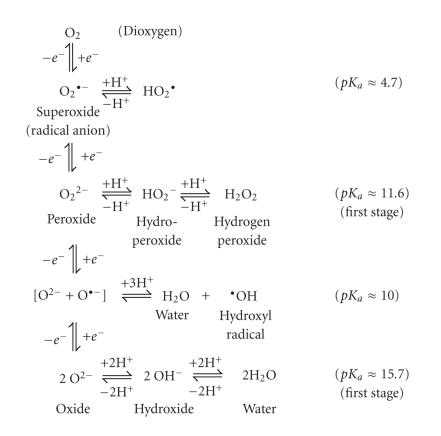
Oxygen redox equilibria.

As shown above, the oxidation of pyrogallol through Trazt-Schorigin reaction is proceeding in the presence of various oxygen species stable in alkaline solution (O_2_, HO_2_^−^, O_2_^−^). However, in the complex regime of pyrogallol oxidation and the intensely reactive environment of the reaction mixture, other oxygen redox state may be involved. Figure 4 shows the redox oxygen equilibria with the H_2_O_2_ species associated with pH values of aqueous media.

### 1.5. Instrumentation (Figure 5)

Recent progress in the field of photomultiplier technology led to the production of highly sensitive detectors; thus, CL-emission of relatively low intensity is now measured accurately. This made CL-detection a useful detecting tool for the determination of *μ*M and even nM concentrations of analytes.

Combining CL-detection with flow-injection manifold, the analytical procedure becomes rapid, convenient, and reproducible; furthermore, the apparatus is simple, inexpensive, and portable for in situ measurements. Finally, it is easily automated for routine analysis work, remote controlled for unattended monitoring.

Interference from other compounds in the sample is confronted by various separation methods or with use of in-line enzyme reactors in most cases.

## 2. PUBLISHED WORKS FROM THE RESEARCH GROUP OF PROFESSOR EVMIRIDIS

A study [7] of the effect of active oxygen species on the pyrogallol oxidation by H_2_O_2_ at pH 11 (optimum condition for CL-emission) was investigated using specific trapping agents and scavengers for each oxygen active species such as NaN_3_ for singlet oxygen, SOD (superoxide dismutase) for the ^•^O_2_^−^, CAT (catalase) and PER (peroxidase) for H_2_O_2_ according to the following reaction:

(3) $$\begin{array}{c} 2{\text{N}}_{3}{}^{-}+{}^{1}{\text{O}}_{2}\to {\text{N}}_{2}\text{O}+\text{NO},\\ 2^{•}{\text{O}}_{2}{}^{-}+2{\text{H}}^{+}\overset{\text{SOD}}{\to }{\text{H}}_{2}{\text{O}}_{2}+{\text{O}}_{2},\\ 2{\text{H}}_{2}{\text{O}}_{2}\overset{\text{CAT}}{\to }2{\text{H}}_{2}\text{O}+{\text{O}}_{2},\\ 2{\text{H}}_{2}{\text{O}}_{2}\overset{\text{PER}}{\to }2{\text{H}}_{2}\text{O}+{\text{O}}_{2} \end{array}$$

**Figure 6:**
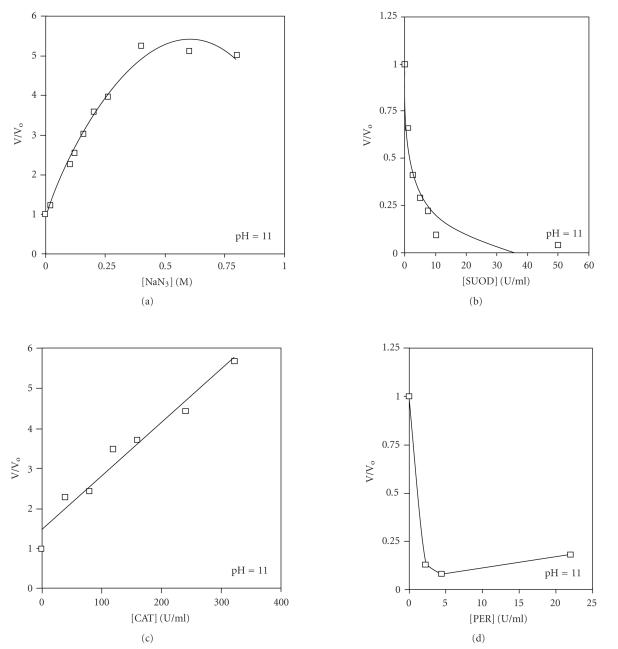
Effect of concentration of scavenger on CL-emission generated during pyrogallol, oxidation by hydrogen peroxide at pH = 11.0: (a) NaN_3_, (b) SUOD, (c) CAT, (d) PER. Conditions: [hydrogen peroxide] = 10 mM and [pyrogallol] = 1 mM.

From the data obtained (Figure 6) it was concluded that ^•^O_2_^−^ species are playing the major role for the appearance of CL-emission at pH 11 since the CL-emission was extinguished by the addition of SOD. In addition, −OOH species are playing role in the CL-emission at lower pH values since the CL-emission increased by the addition of PER at pH 7. Surprisingly, the CL-emission increased with NaN_3_ addition suggesting that NO_X_ species may be involved.

**Figure 7:**
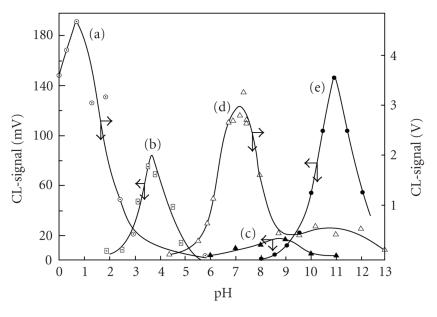
Effect of pH on CL-emission generated during pyrogallol oxidation by oxidant agents: (a) MnO_4_^−^; (b) Ce^4+^; (c) ClO^−^; (d) IO_4_^−^; (e) H_2_O_2_. Conditions: [Pg] = 0.5 mM and [oxidant] = 10 mM.

**Figure 8:**
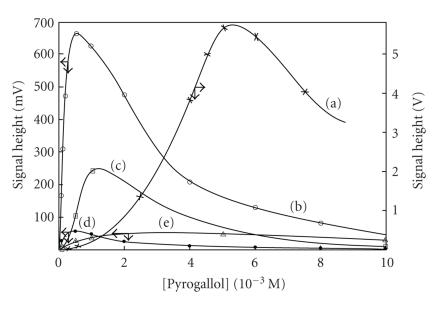
Effect of [pyrogallol] on CL-emission for the oxidant agents: (a) MnO_4_^−^ at pH = 0.65; (b) IO_4_^−^, pH = 8.0; (c) H_2_O_2_, pH = 11.0, (d) Ce^4+^, pH = 3.5; (e) ClO^−^, pH = 9.0, [oxidant] = 10 mM.

The intensity of CL-emission generated during pyrogallol oxidation by different oxidizing agents was followed by various oxidizing reagents [8]. The oxidizing reagents that generated CL-emission at a measurable level of intensity were the permanganate anion with maximum at pH 1.5, Ce(IV) with maximum at pH 4.0 (restricted by precipitation above pH 4), periodate with maximum at pH 7.8, hypochlorite with maximum at pH 9.0, and H_2_O_2_ with maximum at pH 11.0 (Figure 7). The intensity of CL-emission was followed also by the concentration ratio between the oxidizing agent and the pyrogallol at the maximum pH for each oxidant. The ratio obtained when the change of the CL-emission level reached a plateau for the different oxidizing agents was 2 (10 electron oxidation) for the permanganate anion, 7.0 for Ce(IV), 3 (6 electron oxidation for the rapid part of reaction) for periodate anion, and 2 for ClO^−^ and H_2_O_2_; thus suggesting that permanganate anion and Ce(IV) cation are strong oxidizing agents while hypochlorite and hydrogen peroxide are soft oxidants and periodate is in the middle of the scale. Finally, the intensity of CL-emission was followed with pyrogallol concentration and the data obtained (Figure 8) showed that the maxima for CL-emission were 5 mM for permanganate anion, 0.5 mM for the Ce(IV) cation, 0.75 mM for periodate anion, around 3 mM for hyprochlorite anion, and around 1.5 mM for H_2_O_2_. The order of sensitivity based on optimum conditions for each oxidizing agent is MnO_4_^−^ ≫ IO_4_^−^ > H_2_O_2_ > Ce(IV) ≈ ClO^−^.

The possibility for finding compounds with enhancing effect was tested and among the ones tested the NH_2_OH · HCl increased CL-emission generated during the pyrogallol oxidation by periodate 6-fold [9]. Furthermore, the CL-emission was followed with the concentration ratio between the pyrogallol and the hydroxylamine hydrochloride and an optimum ratio was found to be around 1. From absorption spectra and fluorescence spectra, the formation of Pyrogallol-Hydroxylamine complex was indicated [10].

**Figure 9:**
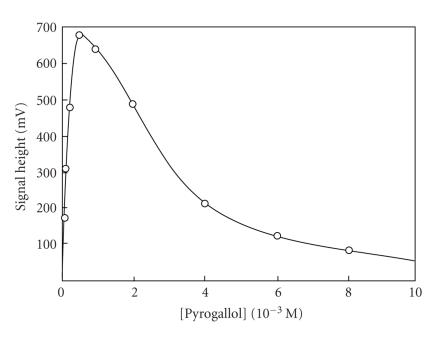
CL-intensity versus [pyrogallol] with merging zone mode of operation. Conditions: [periodate] = 10 mM; pH = 8.0.

**Figure 10:**
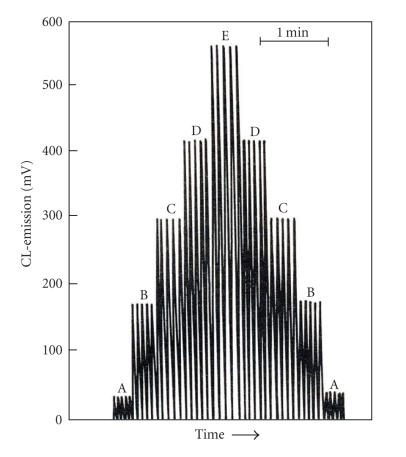
Typical recorder outputs for periodate-pyrogallol-system. Conditions: [periodate] = 10 mM; pH = 8.0. Pyrogallol concentrations: (A) 0.1 mM; (B) 0.5 mM; (C) 1.0 mM; (D) 2.0 mM; (E) 10 mM.

Optimized conditions for the determination of pyrogallol based on CL-emission generated during the periodate oxidation in the presence of hydroxylamine hydrochloride using FI/CL-detection apparatus were found, and an analytical method for pyrogallol determination was developed [11] (Figure 9). The reported analytical parameters are linear valid range from 5 *μ*M up to 0.75 mM; sensitivity 270 V L/mol; LOD 1.0 *μ*M; RSD 1%, and sample throughput 10 per minute (Figure 10).

Optimized conditions were obtained for the determination of periodate based on CL-emission [12] generated by pyrogallol using flow-injection/CL-detection apparatus. A sigmoid calibration curve was obtained with periodate standard solutions in the range of 0.5 mM up to 10 mM with LOD 10 *μ*M, RSD 3%, and sample throughput 15 per minute.

**Figure 11:**
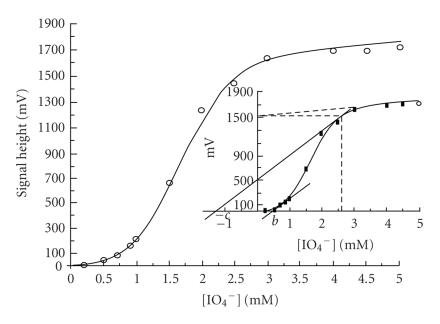
Fit of model equation. The small scale graph is drawn to demonstrate the method of approximating the parameters' starting values. Conditions: [Pg] = 1.0 mM; [Hx] = 1.0 mM; pH = 8.0.

A procedure [13] of the best fit model equation of periodate calibration curve followed by parameterization of the equation constants was based on home software using a modification of the pattern search method and supported by a graphical method for approximating the initial parameter values of each model equation tested and criteria based on absolute error of residual variance. Among the various model equations, a best fit to the experimental data was found with the model equation

(4) $$y=ax^{2}+c\sqrt{x}+1$$

with parameters *a* = 39 ± 5, *b* = 0.5 ± 0.2, and *c* = 1.3 ± 0.1 (Figure 11).

A study [14] of the error structure of the model equation $y=ax^{2}+c\sqrt{x}+1$ parameters was studied using the pattern search modification (mentioned above) for optimum parameter estimation based on various criteria (absolute or relative error) of residual variance, assuming normal distribution of errors and outliers, and employing a pseudorandom number generator of the mixed congruential method for collecting errors from a normal distribution of population errors and outliers according to the procedure described. Absolute errors or relative errors were considered. The results obtained were tested for the percentage of bias and the distributions of the errors for skewness and excess curtosis.

**Figure 12:**
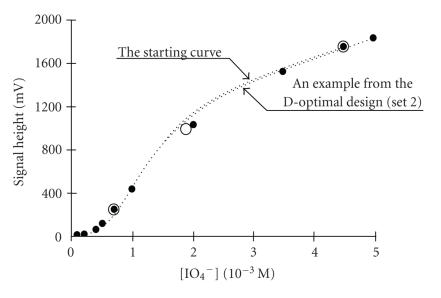
Periodate calibration curve drawn from initial and optimal-design points indicated in the figure by arrows; (•) ten experimental points. Conditions: [Pg] = 1.0 mM; [Hx] = 1.0 mM; pH = 8.0.

In another study the experimental design is obtained for the rapid estimation of the parameters of the sigmoid periodate calibration curve based on the D-optimality criterion [15]. With this facility it was not necessary to find a large number of data points to draw the sigmoid periodate calibration curve (Figure 12).

The determination of periodate by flow-injection/CL-detection method [12] was applied for the indirect determination of the ethylene glycol by measuring the excess of periodate remained after treatment of the sample with specific volume of standard periodate solution. The proposed method was applied for the determination of ethyleneglycol in real samples such as commercial car-antifreeze preparations and water from car radiators and the proposed method was in close agreement with Fleury-Lange method used as a validation test (Table 1).

A simulaltaneous kinetic determination of glucose and fructose was proposed [16] using the flow-injection CL-detection method in samples containing both analytes in mixture. The proposed method is based on the difference in kinetic constants of periodate oxidation between these two analytes. The experimental data from the standard solutions of mixed analytes in different proportions are found to follow the kinetic equation

(5) $${\left[{\text{IO}}_{4}{}^{-}\right]}_{t}={\left[{\text{IO}}_{4}{}^{-}\right]}_{0}-5{\left[\text{Hex}\right]}_{0}\left\{1+\frac{L}{{\left[{\text{IO}}_{4}{}^{-}\right]}_{0}e^{-kLt}-5{\left[\text{Hex}\right]}_{0}}\right\}$$

and the periodate calibration curve equation

(6) $$\text{CL}\left(\text{mV}\right)=\frac{a{\left[{\text{IO}}_{4}{}^{-}\right]}_{t}^{2}}{b{\left[{\text{IO}}_{4}{}^{-}\right]}_{t}-c\sqrt{{\left[{\text{IO}}_{4}{}^{-}\right]}_{t}}+1}$$,

where *L* = 5[Hex]_0_ − [IO_4_^−^]_0_ and [Hex]_0_ = [Glc]_0_ + [Fru]_0_, [Glc]_0_ = initial glucose concentration in the sample, [Fru]_0_ = initial fructose concentration in the sample, *k* = overall kinetic constant, time from the start of oxidation reaction.

**Figure 13:**
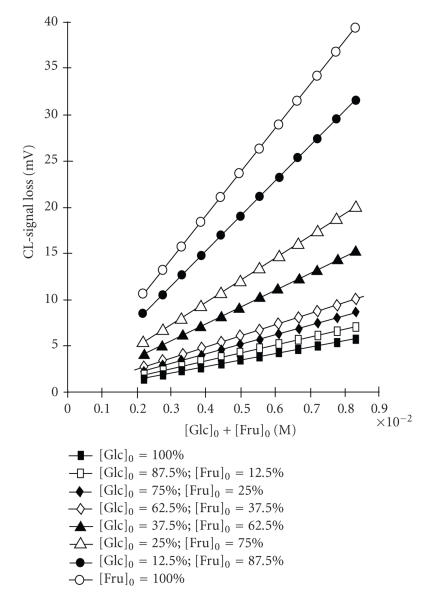
Curves of CL-emission loss versus [Hex]_0_ at various ratios of [Glc]_0_/[Fru]_0_ at calculated from initial rates. Conditions: the reagent solution is 1.0 mM pyrogallol/1.0 mM NH_2_OH · HCl in buffer solution of pH = 8.0 (0.5 M NaHCO_3_/1.0 M HCl) and the carrier stream is a buffer of pH = 8.0.

The overall kinetic constant is changed with the ratio of [Glc]_0_/[Fru]_0_ at constant [Hex]_0_ exponentially. Calibration curves with concentration of [Hex]_0_ at various constant ratios of [Glc]_0_/[Fru]_0_ are shown in Figure 13.

**Figure 14:**
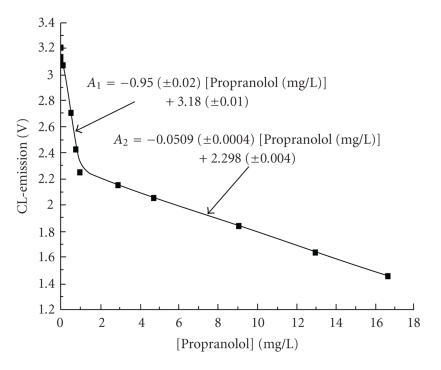
Propranolol calibration curve for both concentration ranges under optimal conditions. ([Pg] = 1.0 mM, [Hx] = 1.0 mM, [IO_4_^−^] = 2.5 mM, pH = 8.02, reaction time: 7 min, flow rate: 1.71 mL min^−1^, sample volume: 100 *μ*L, PMT voltage: 1400 kV).

The indirect determination of propranolol with periodate oxidation in a flow-injection/CL-detection apparatus is proposed [17] sed on the determination of the excess of periodate. The calibration curve follows two linear ranges (Figure 14), the initial one is short and sharp and covers the concentration range 0.10 to 1.0 ppm with LOD 37 ppb and RSD 0.4%; the second one covers the concentration range 1.0 to 20 ppm and with sensitivity 20 times less than the first one. In addition, a study for interferences was made that showed NO^−^_2_ and Cr^3+^ to be the major interferent compounds (Table 2).

**Figure 15:**
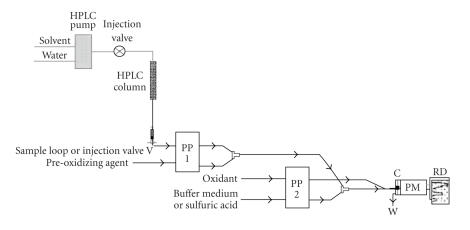
RP-HPLC apparatus implemented by post-column Flow-injection/CL-detection apparatus set-up.

A study of the CL-emission generated by oxidation of pyrogallol was made on-line with HPLC of eluted analytes [18, 19] using post-column FI/CL-detection apparatus (Figure 15).

RP-HPLC elution solvents include MeOH, AcCN, and THF in mixtures with water. In general, CL-emission is generated by permanganate oxidation of organic compounds. The CL-emission generated by the permanganate oxidation of organic solvent gives an intense background that makes CL-emission of eluted analytes undetectable.

**Figure 16:**
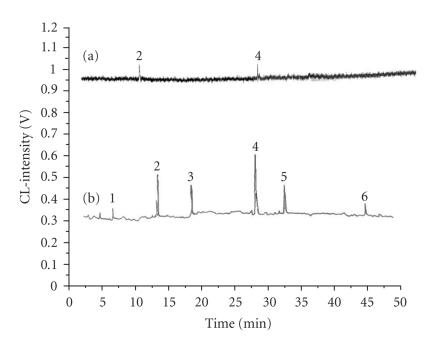
HPLC-CL-detection isocratic chromatogram of six UV-adsorbing compounds used for sun protection preparations. (a) Direct acidic permanganate CL-detection, (b) SPOC (solvent pre-oxidative chemiluminescence) periodate-direct acidic permanganate CL-detection. Flow rates: FIA-SPOC = 2.5 mL min^−1^ and FIA-CL = 2.2 mL min^−1^, HPLC = 1.0 mL min^−1^, [KMnO_4_] = 5 mM, [H_2_SO_4_] = 5 M, [KIO_4_] = 10 mM. Peak assignment: (1) E232, (2) Bz-3, (3) E6300, (4) PABA, (5) E2292, and (6) E9020.

**Figure 17:**
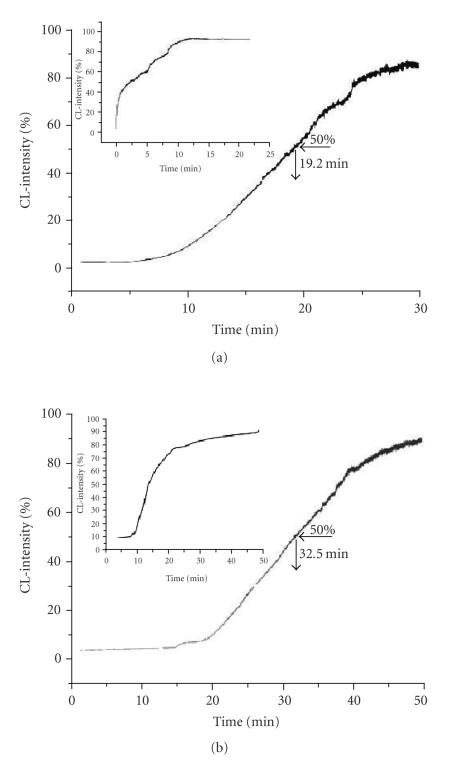
RP-HPLC/SPOC baseline profile during gradient elution (a) 0–100% (v/v) MeOH, *t* = 30 min, (b) 0–100% (v/v) MeOH, *t* = 45 min.

In both these research studies, attempts are made to reduce the background level. The studies resulted in the method of solvent pre-oxidative chemiluminescence (SPOC) that decreases the background level, adequately for the determination of all the sunlight absorbing ingredients of a commercial preparation in RP-HPLC isothermal elution (Figure 16) and to obtain a smooth slow increase of background in gradient elution (Figure 17).

## 3. PUBLISHED RESEARCH REPORTS FROM OTHER AUTHORS

The oxidation of H_2_O_2_ by periodate in potassium carbonate aqueous solution was employed by Lin et al. [20] for the determination of trace amounts of H_2_O_2_ in snow water with flow-injection/CL-detection apparatus. The ratio of the signal-to-noise (S/N) is proportional to the concentration of hydrogen peroxide up to 10 *μ*M. The detection limit with the flow-injection method is 0.005 *μ*M H_2_O_2_ (S/N = 3). The relative standard deviation (RSD) for 0.04 *μ*M hydrogen peroxide is 2.8% (*n* = 14). Sample throughput is ca. 100 h^−1^. The selectivity of this method is very high, and most of the transition metal ions have no effect on the determination.

A sensitive and stable periodate-hydrogen peroxide CL-system was used by Zhang and Chen [21] for the determination of sodium dodecyl benzene sulfonate in a flow-injection/CL-detection apparatus. The 3*σ* detection limit for SDBS is 0.032 *μ*g/mL in a system using a trace of cyclohexane and the relative standard deviation of this method is 1.6% at 1.0 *μ*g/mL for 11 determinations.

A flow-injection chemiluminescent method is proposed by Nakano et al. [22] for the determination of vanadium (IV) and total vanadium by its catalytic action on the periodate-purpurogallin CL-reagent to generate light at 4^°^C. The presence of hydrogen carbonate enhanced the CL-emission arising from the vanadium (IV)-catalyzed reaction. Vanadium (V) is then determined after being reduced to vanadium (IV) by using an on-line silver reducing column. Calibration curves for vanadiums (IV) and (V) were linear in the range 0.1–10 ng/mL with sampling rate of about 50 h^−1^. The limit of detection for signal-to-noise ratio of 2 was 0.05 ng/mL and the relative standard deviations were 1.4 and 1.6% for ten determinations of 2.0 ng/mL of vanadiums (IV) and (V), respectively. Interferences from metal ions could be eliminated by the use of O,O′-bis(2-aminoethyl)ethyleneglycol-N,N,N′,N′-tetraacetic acid and diphosphate as masking agents. The proposed method was successfully applied to the determination of vanadium (IV) and total vanadium in fresh water samples.

A flow-injection/CL-sensor apparatus is described by Xiong et al. [23] for the determination of isoniazid in urine samples. The sensor is based on molecularly imprinted polymer technology and the detection of luminol-periodate CL-reagent. The enhanced CL-intensity is linear in the range 0.002–0.2 *μ*g/mL and the detection limit is 0.007 *μ*g/mL (3*σ*) isoniazid with a relative standard deviation 2.8% (*n* = 9) for 0.08 *μ*g/mL. The sensor is reversible and reusable. It has a great improvement in sensitivity and selectivity for CL-analysis. As a result, the sensor has been successfully applied to determination of isoniazid in human urine. At the same time, the binding characteristic of the polymer to isoniazid was evaluated by batch method and the dynamic method, respectively.

## Figures and Tables

**Figure 5:**
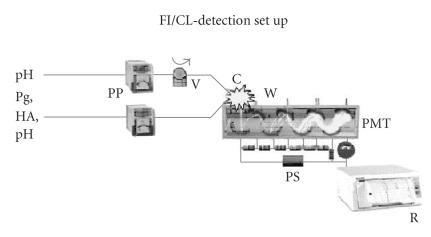
Flow-injection/CL-detection set up for the studies of CL-emission performed in analytical chemistry chemiluminescence research laboratory of Chemistry Department in University of Ioannina; PP = peristaltic pump, V = sample injection valve, C = snail-shell-like cell, PMT = photomultiplier, PS = power supply, R = recorder, W = waste.

**Table 1:** Validation of the proposed method with standard method for determination of ethylene glycol content in car-antifreeze commercial products.

Sample	Ethylene glycol content, mg/mL	

	Fleury-Lange^*^ method	CL-detection^†^ method

*Ethylene glycol solutions*		
A	2.79	2.79
B	3.26	3.26
C	3.72	3.63
D	4.06	4.03

*Antifreeze solutions*		
BP	3.30	3.40
Glycoshell	3.35	3.53

*Car radiator water*		
One-year-old car	0.336	0.372
Six-year-old car^§^	0.458	0.418
Seventeen-year-old car^††^	0.031	0.062

*RSD 2%.

^†^RSD 3%.

^§^Radiator content renewed in previous year.

^††^Car radiator with tiny leaks.

**Table 2:** Propranolol-interferant proportions used for the interference study.

*Interferant*	Propranolol: interferant proportion

Glucose	1 : 100
Fructose	1 : 100
Citric acid	1 : 100
Starch	1 : 100
Sucrose	1 : 100
Urea	1 : 100
Ascorbic acid	1 : 50
MoO_4_ ^2−^	1 : 100
SO_3_ ^2−^	1 : 50
NO_2_^−^	1 : 5
Na^+^	1 : 100
K^+^	1 : 100
Mg^2+^	1 : 100
Ca^2+^	1 : 100
Fe^3+^	1 : 100
Cr^3+^	1 : 5
